# A family cluster of three confirmed cases infected with avian influenza A (H7N9) virus in Zhejiang Province of China

**DOI:** 10.1186/s12879-014-0698-6

**Published:** 2014-12-31

**Authors:** Hua Ding, Yin Chen, Zhao Yu, Peter W Horby, Fenjuan Wang, Jingfeng Hu, Xuhui Yang, Haiyan Mao, Shuwen Qin, Chengliang Chai, Shelan Liu, Enfu Chen, Hongjie Yu

**Affiliations:** Hangzhou Centre for Disease Control and Prevention, Hangzhou, Zhejiang Province China; Zhejiang Provincial Centre for Disease Control and Prevention, Hangzhou, Zhejiang Province China; Oxford University Clinical Research Unit-Wellcome Trust Major Overseas Programme, Hu Chi Minh City, Vietnam; Centre for Tropical Medicine, Nuffield Department of Clinical Medicine, Oxford University, Oxford, United Kingdom; Singapore Infectious Disease Initiative, Singapore City, Singapore; XiaoShan District Centre for Disease Control and Prevention, Hangzhou, Zhejiang Province China; ShangCheng District Centre for Disease Control and Prevention, Hangzhou, Zhejiang Province China; Division of Infectious Disease, Key Laboratory of Surveillance and Early-warning on Infectious Disease, Chinese Center for Disease Control and Prevention, No. 155 Changbai Road, Changping District, Beijing, China; Department of Infectious Diseases, Zhejiang Provincial Center for Disease Control and Prevention, 3399 Binsheng Road, Binjiang District, Hangzhou, 310051 Zhejiang China

**Keywords:** H7N9 subtype, Family cluster, Live bird market, Death, Epidemiological investigation

## Abstract

**Background:**

A total of 453 laboratory-confirmed cases infected with avian influenza A (H7N9) virus (including 175 deaths) have been reported till October 2,2014, of which 30.68% (139/453) of the cases were identified from Zhejiang Province. We describe the largest reported cluster of virologically confirmed H7N9 cases, comprised by a fatal Index case and two mild secondary cases.

**Methods:**

A retrospective investigation was conducted in January of 2014. Three confirmed cases, their close contacts, and relevant environments samples were tested by real-time reverse transcriptase-polymerase chain reaction (RT-PCR), viral culture, and sequencing. Serum samples were tested by haemagglutination inhibition (HI) assay.

**Results:**

The Index case, a 49-year-old farmer with type II diabetes, who lived with his daughter (Case 2, aged 24) and wife (Case 3, aged 43) and his son-in-law (H7N9 negative). The Index case and Case 3 worked daily in a live bird market. Onset of illness in Index case occurred in January 13, 2014 and subsequently, he died of multi-organ failure on January 20. Case 2 presented with mild symptoms on January 20 following frequent unprotected bed-side care of the Index case between January 14 to 19, and exposed to live bird market on January 17. Case 3 became unwell on January 23 after providing bedside care to the Index case on January 17 to 18, and following the contact with Case 2 during January 21 to 22 at the funeral of the Index case. The two secondary cases were discharged on February 2 and 5 separately after early treatment with antiviral medication. Four virus strains were isolated and genome analyses showed 99.6 ~100% genetic homology, with two amino mutations (V192I in NS and V280A in NP). 42% (11/26) of environmental samples collected in January were H7N9 positive. Twenty-five close contacts remained well and were negative for H7N9 infection by RT-PCR and HI assay.

**Conclusions:**

In the present study, the Index case was infected from a live bird market while the two secondary cases were infected by the Index case during unprotected exposure. This family cluster is, therefore, compatible with non-sustained person-to-person transmission of avian influenza A/H7N9.

**Electronic supplementary material:**

The online version of this article (doi:10.1186/s12879-014-0698-6) contains supplementary material, which is available to authorized users.

## Background

Human infection with avian influenza A/H7N9 virus was first identified in March 31 of 2013, in China, a total of 453 confirmed cases were found in the world up to date [1]. The seasonal epidemiology is characterized to occur from November through April in China, coinciding well with both seasonal human influenza and H5N1 in birds [2]. Almost all cases were hospitalized, and 1/3 of cases died. The fatality is much higher than that for seasonal influenza in the China (0.04%), but it is lower than for cases of H5N1 (60%) [3],[4]. Current evidence suggests that human infection appears to be associated with exposure to infected live poultry or contaminated environments, including markets where live poultry are sold [5]-[7]. In the light of this opinion, the closure of live bird markets (LBM) has been associated with a reduction in the incidence of human infections [8]. Despite the fact that H7N9 remains to be a zoonotic infection of avian origin, there are concerns that the virus show genotypic and phenotypic evidence of partial adaptation to mammals [9]. Compared to other subtypes of avian influenza virus, H7N9 virus show increased binding affinity to mammalian-type receptors, and their amount grow up rapidly at the temperatures that are close to the normal body temperature in mammals (although it is lower than that of birds). In addition, they possess PB2 gene mutations that are associated with adaptation to mammals [10]-[12]. Whilst sequence analyses had shown that the haemagglutinin (HA) and neuraminidase (NA) genes of H7N9 virus detected in China show very high homology, whereas the genes for coding internal proteins are diversified [13]. Ferret and mouse models confirm that strains isolated from humans could replicate efficiently in both mammalian and human airway cells, with efficient transmissibility by direct contact and modest transmissibility by respiratory droplets [14],[15]. Given these signatures of partial adaptation to mammals, it is imperative to closely monitor and investigate all clusters of human H7N9 virus to determine the transmissibility and severity of virus infection, as well as its potential host and pathogen determinants.

A few of family clusters of H7N9 infections (in Shanghai, Jiangsu, Shandong, Guangdong and Beijing) have been described involving two family members. It was concluded that limited person-to-person transmission may occur following close, prolonged, and unprotected contact with the symptomatic Index case, while sustained transmission was not found [16]-[18]. Here we describe an additional cluster, comprised of three laboratory-confirmed cases of human infection with H7N9 virus reported in Zhejiang Province in January 2014. This is the largest reported cluster of virological confirmed H7N9 cases, and the full genome data of the virus were isolated from all cases and associated with clinical and epidemiological data and their close contacts.

## Methods

### Informed consent and ethical approval

All three H7N9 confirmed cases and 25 adult contacts and surveillance cases had provided written consent for the participation in this study and the publication of their individual details. Data collection for H7N9 cases was determined by the National for H7N9 cases was determined y f man of China, as a part of the continuing public health outbreak investigation; therefore, it was exempt from assessment by institutional review board. The protocol for collecting epidemiological data and conducting serological test of close contacts were approved by the institutional review board of the China CDC.

### Case ascertainment

Suspected cases of human infection with H7N9 virus are identified through the Chinese surveillance systems for influenza-like illness, severe acute respiratory illness (SARI), pneumonia of unexplained origin, and clinical diagnostics of cases of pneumonia. Based on the Chinese guidance, an individual could be considered as a confirmed case of H7N9 virus infection if the presence of the H7N9 virus is verified by real-time reverse transcriptase polymerase chain reaction assay (RT-PCR), virus isolation, or serologic testing [19].

### Cases and procedures

Epidemiological and clinical data were collected through interviews and reviews of medical records between January 13 and 25, 2014. All three cases and their relatives were interviewed by public health staff to record their exposure history during the two weeks before the onset of symptoms, to validate the timeline of events and to identify close contacts.

Respiratory tract samples were collected from the Index case, Case 2, and Case 3, on January 18, 22, and 23, respectively. Environmental samples were collected from the LBM (A1 market) and the secondary wholesale markets (B1, C1, and D1 markets) and from a neighboring household where several chickens were bred. All samples were placed in sterile viral transport medium and shipped within 24 hours to the laboratory of Zhejiang CDC at 4°C for H7N9 testing.

Viral RNA was extracted using Qiagen RNeasy Mini Kit. Real-time RT-PCR was used to detect influenza type A, subtype H7 and N9 using the protocol, specific primer and probe sets provided by China CDC [20]. Specimens were also tested by RT-PCR for the presence of seasonal influenza virus (H1, H3, and B) and H5N1 virus. Complete genomic fragments of the H7N9 virus were amplified directly from clinical samples, and sequencing was performed using an ABI 3730XL automatic DNA analyzer. The nucleotide sequences were determined by dideoxy sequencing using an ABI Prism BigDye Terminator cycle sequencing kit as previously described [21]. Nucleotide sequences were analyzed with the DNASTAR package (Lasergene, Madison, WI, USA). Phylogenetic analysis was done by neighbor-joining method with MEGA (version 5.2).

Close contacts were placed under daily active surveillance for fever and respiratory symptoms, which was last for seven days after their last exposure to the H7N9-infected case. Close contacts were defined as individuals who had close contact (<1 meter) with any case without the use of personal protective equipment at any time before illnesses onset to the time of isolation of the case in hospital. Antiviral chemoprophylaxis was neither recommended nor provided to contacts.

Following written informed consent, a structured questionnaire was used to gather demographic information and data on use of personal protective equipment, antiviral chemoprophylaxis, symptoms, and potential risk factors for H7N9 infection during the two weeks starting from their last exposure to H7N9-infected cases.

Respiratory specimens for H7N9 testing were taken from close contacts with a febrile respiratory illness occurred during the 7-day observation period. Contacts were asked to provide a single convalescent serum collected ≥ 3–4 weeks after their last exposure to a case with H7N9. H7N9 serological testing was done by HI assay using a modified horse red-blood-cell assay, recommended by the WHO. The antigen used for the HI assays was the A/Zhejiang/1/2013(H7N9) strain. A HI titer ≥ 1:40 in single serum sample and a four-fold or greater rise in titer in paired sera was defined as seropositive.

## Results

### Clinical features of three confirmed cases

**Figure 1:**
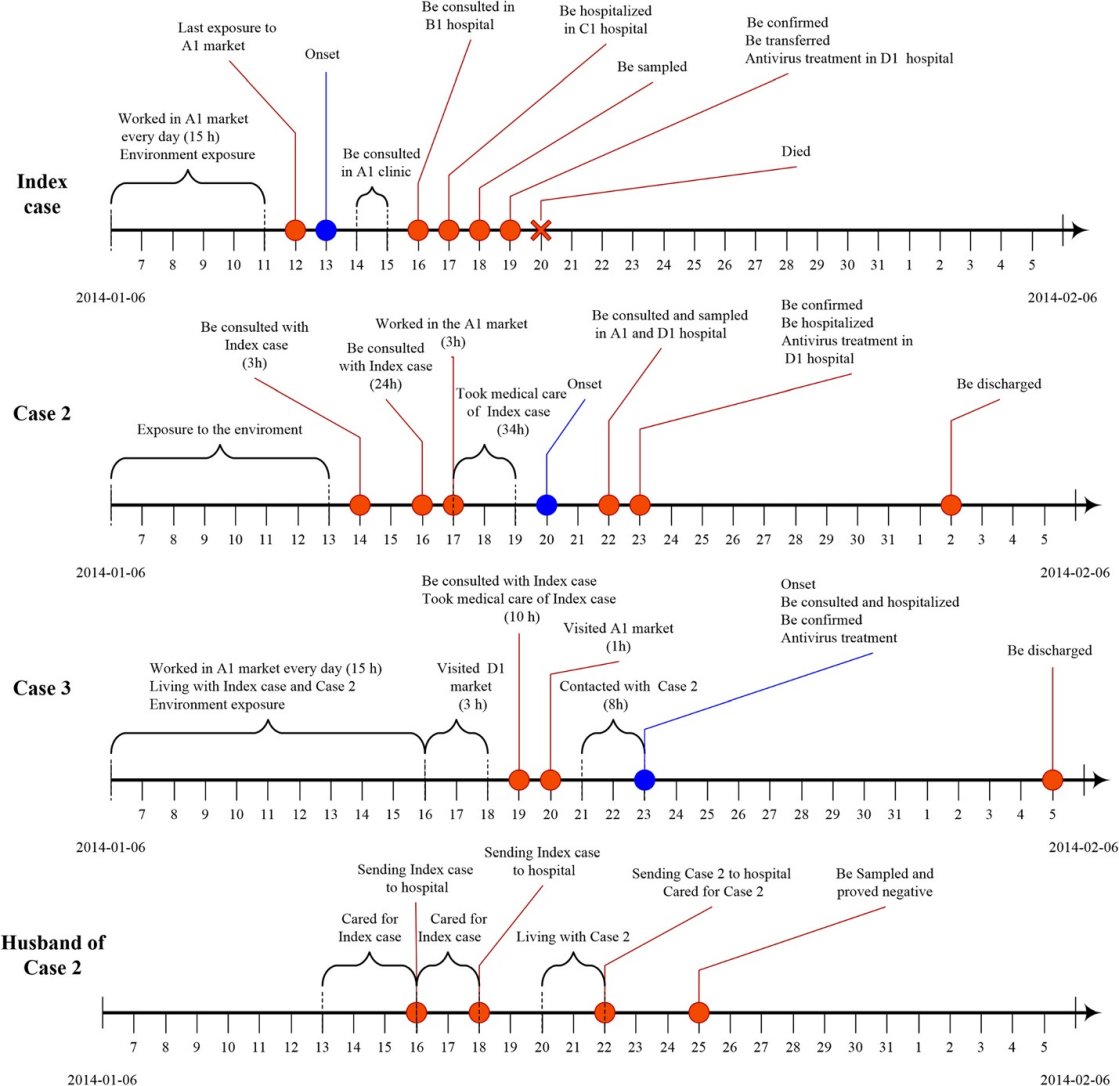
**Illustrates the progression for the three cases with confirmed H7N9 infection and one close contact.**

**Figure 2:**
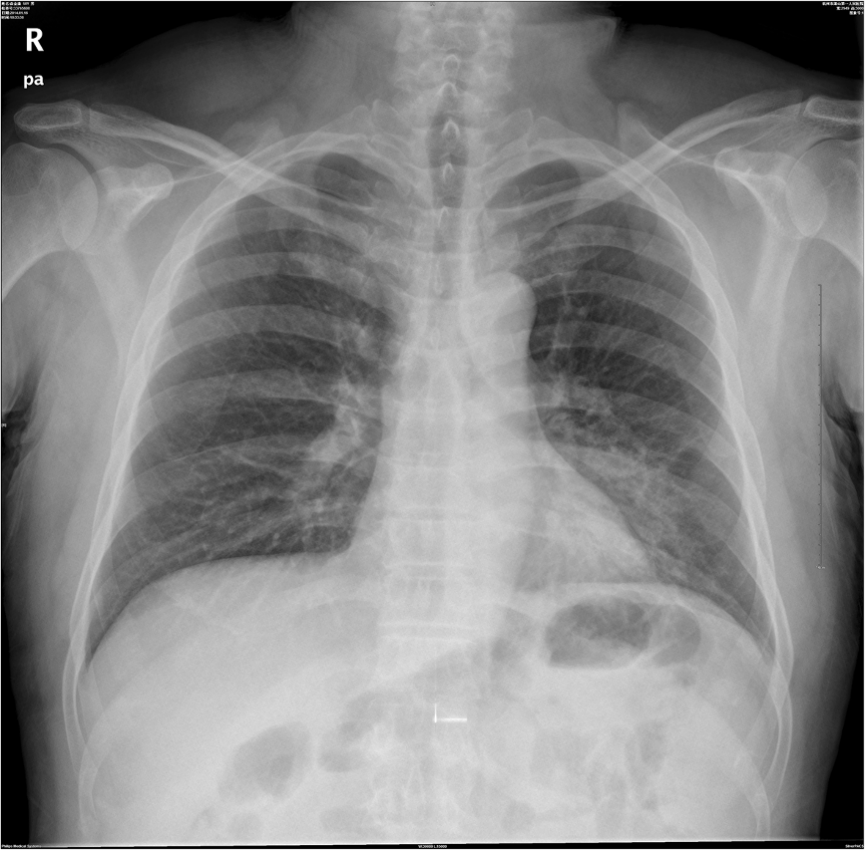
**Chest radiograph of the Index case on three days of illness.**

The Index case, a 49-year-old farmer with type II diabetes, taking antidiabetic drugs for one year, had been unwell since January 13, 2014, with fever (39.6°C) and flank pain. After consulting a health care clinic (A1 clinic) on January 14 and 15, he was treated as an outpatient with Ciprofloxacin and intravenous Amoxicillin/Clavulanate potassium. On January 16, he made a further consultation at a local hospital (B1 hospital) owing to persistent fever. Chest radiography showed a left-lower-lobe pneumonia; meanwhile, treatment with Ciprofloxacin was continued. Peripheral blood cell count was normal. On January 17, Index case’s condition was worsened and again medical advice was sought; therefore, Index case was admitted to a different hospital (hospital C1). Upon admission in hospital C1, he had severe leucopenia, lymphopenia and thrombocytopenia (Table 1). He was diagnosed with community acquired pneumonia with a left pleural effusion. On January 18, he consulted at hospital D1 (a more advanced hospital) where a sputum sample was collected and sent to Zhejiang CDC for microbiologic testing. On January 19, H7N9 virus-specific RNA was detected by RT-PCR (Ct value 29) in the sputum sample. Once the H7N9 virus infection was confirmed, the patient was transferred from hospital C1 to D1 immediately. At hospital D1, he was isolated in a single room, where he was intubated, mechanically ventilated and commenced on Oseltamivir (75 mg, twice daily by nasogastric tube) and Peramivir (600 mg, once daily, intravenously). On January 20, the patient died of acute respiratory distress syndrome (ARDS) and multi-organ failure (Table 1, Figure 1, Figure 2, and Additional 1: Figure S1).

**Table 1:** **Clinical features of the three confirmed H7N9 cases on admission**

Findings	Index case	Case 2	Case 3	Normal value
General information				
Age (years) and sex	49, Male	24, Female	43, Female	
Temperature (°C)*	40	39.5	36.8	37.0
Blood counts*				
White blood cells (×10^9^ per L)	1.8	6.1	7.6	4.0 ~ 10.0
Lymphocytes (×10^9^ per L)	0.2	0.8	1.1	0.8 ~ 4.0
Platelets (×10^9^ per L)	89	157	168	101 ~ 320
Serum biochemistry*				
Alanine aminotransferase (U/L)	76.8	24	40	8 ~ 40
Aspartate aminotransferase (U/L)	63	40	35	8 ~ 40
Lactate dehydrogenase (U/L)	435	188	110	109 ~ 245
Creatine kinase (U/L)	324.5	66	50	20 ~ 140
C-reactive protein (mg/L)	94	9.0	6	0 ~ 8
Arterial blood*				
PaCO_2_ (mm Hg)	17	39	26	35.0 ~ 45.0 mmHg
PaO_2_ (mm Hg)	69	165	85	83 ~ 108 mmHg
Blood oxygen saturation (%)	75	99	97	95 ~ 98%
Chest radiography	Bilateral lower-lobe infiltrate and left pleural effusion	Normal	Normal	
Complication	ARDS and multi organ failure	No	No	
Treatment				
Mechanical ventilation	Yes	No	No	
Antiviral treatment	Oseltamivir (75 mg Bid po) + Peramivir (600 mg Qd iv)	Oseltamivir (75 mg Bid po) + Peramivir (300 mg Qd iv)	Oseltamivir (75 mg Bid po) + Peramivir (300 mg Qd iv)	
	(Commenced 6 days after illness onset)	(Commenced 3 days after illness onset)	(Commenced 1 day after illness onset)	
Antibiotic treatment	Yes	Yes	Yes	
Oxygen treatment	Yes	Yes	Yes	
Outcome	Died after 7 days of onset	Survived (mild case)	Survived (mild case)	

*Data are the measurements at admission (peak measurement during hospitalization).

Note: ARDS = Acute respiratory distress syndrome; Bid = twice daily; Qd = Once a day.

Case 2 (Index case’s daughter, Figure 1), a 24-year-old female with no underlying diseases, developed a throat sore and cough on January 20, the day her father died. She initially consulted the healthcare clinic in hospital A1 due to constant fever on January 22, where she was treated with antibiotics (amoxicillin) and then transferred to hospital D1 for further examination, where sputum and throat swab samples were taken. RT-PCR was conducted on the samples of sputum and throat swabs on the January 23, and the influenza A/H7N9 specific RNA (Ct value 34) was positive. Afterwards, Case 2 was admitted directly to an isolation room at the hospital D1 and commenced on oral Oseltamivir (75 mg, twice daily), and intravenous Peramivir (300 mg, once daily). On admission her peripheral blood count, serum blood biochemistry, and chest CT scan were normal (Table 1). She was given supplemental oxygen via nasal cannula with a flow rate of 1–3 L/min, whereas her oxygen saturation was 99%. Her condition remained stable, and symptoms were improved during hospitalization. Later, she was completely recovered and was discharged on February 2 after sputum samples tested negative for H7N9 RNA by RT-PCR on January 30 and February 1 (Table 1, Figure 1).

Case 3 (Index case’s wife and Case 2’s mother), a 43-year-old female farmer, with no underlying diseases, developed an acute cough with expectoration on January 23. She attended the hospital D1 where a throat swab was collected and an RT-PCR assessment was conducted on the throat swab sample, which was positive for H7N9 (Ct value of 37). She was admitted to the hospital D1 on January 23. Although chest radiography was normal, she was treated empirically with oral Oseltamivir (75 mg, twice daily) and intravenous Peramivir (300 mg, once daily). Results of peripheral blood cell count, serum electrolytes, renal and liver function, and coagulation profiles were normal. Arterial blood gas results were normal while the patient was breathing room air. The case remained stable during her admission and then she was discharged on February 5 after sputum tested H7N9 negative by RT-PCR on February 4 (Table 1, Figure 1). The husband of Case 2, who had been in close contact with the Index case and Case 2, had no respiratory symptoms, and throat swabs and paired serum samples were negative (Figure 1).

### Laboratory investigations

**Figure 3:**
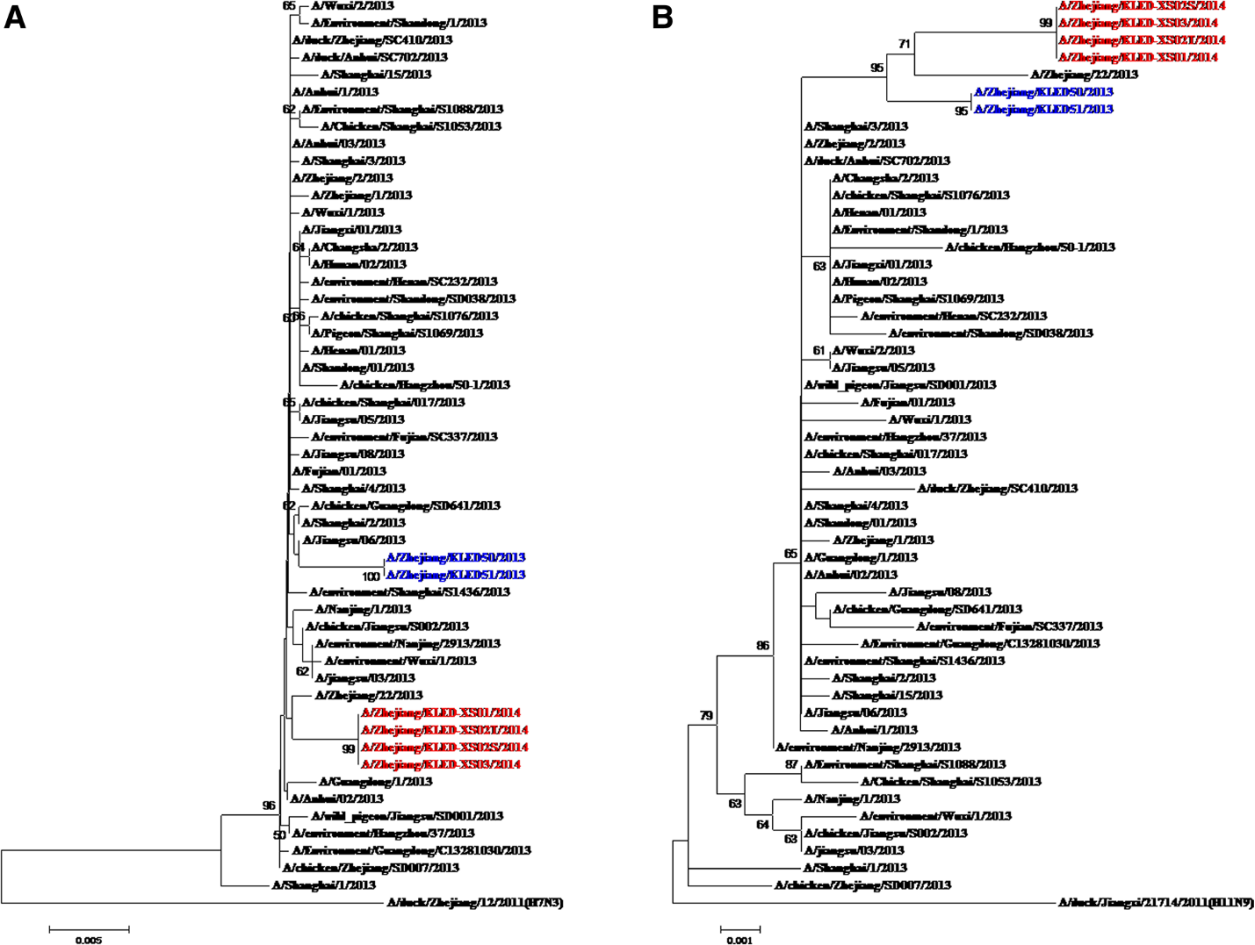
**Phylogenetic analysis of HA and NA segments from the four H7N9 positive samples among three confirmed cases. A**: HA; **B**: NA. Notation: Red color strains noted the isolates from three family cluster cases, 2014 while the blue color noted the isolates from the two sporadic confirmed cases in Zhejiang province, in 2013. A/Anhui/1/2013 was H7N9 vaccine candidate isolate in this 8 segments.

RT-PCR-positive throats swabs or sputum samples were obtained on 5 days, 2 days and 1 day of illness for the Index case, Case 2, and Case 3, respectively. From these samples, four complete full genome sequences were amplified. Sequence analyses indicated that the four isolates were highly homologous the other H7N9 strains previously identified in Shanghai, Jiangsu, Anhui Province, and with candidate vaccine strains (sharing 99.6 ~ 100% identity in amino acid sequences of all 8 segments). The four sequences from the three confirmed H7N9 case shared 96.4 ~ 99.6% homology with the animal isolates (A/chicken/Zhejiang/SD019/2013), and phylogenetic analysis showed that the four isolates were almost genetically identical to other H7N9 virus isolated from the other provinces and chickens. Furthermore, amino acid analyses showed that the HA gene of all four strains possessed the mutation 226 L, indicating high affinity to human receptor alpha 2–6 sialic acid receptors. It showed that the four isolates were entirely of human origin, and NA protein possessed amino acid sequences associated with susceptibility to neuraminidase inhibitor antiviral drugs (H294 and E120 and H276 in NA). The 8 fragments isolated from the Index case, and two secondary cases were identical except for three non-synonymous amino substitutions identified in the Index case. These were G574A nucleic acid substitution (Aa mutation V192I) in the NS gene, G1095A nucleic acid substitution in the PB1 gene (nonsense amino mutation) and C839T nucleic acid substitution (V280A) in the NP gene. (Figure 3, Additional file 1: Figure S2, Table 2 and Table 3).

**Table 2:** **Molecular analysis of three confirmed cases infected with avian influenza A (H7N9) virus, Zhejiang Province, China**

Protein ^*^	Mutations responsible for phenotype change	Position	Family cluster isolates			The human spondaic isolates				H7N9 vaccine candidate isolate	The animal isolates
			Index case	Case 2	Case 3	A/Zhejiang/1/2013	A/Zhejiang/22/2013	A/Zhejiang/01/2014	A/Shanghai/1/2013	A/Anhui/1/2013	A/chicken/Jiangsu/2013
HA	Cleavage site		PEIPKGR*G	PEIPKGR*G	PEIPKGR*G	PEIPKGR*G	PEIPKGR*G	PEIPKGR*G	PEIPKGR*G	PEIPKGR*G	PEIPKGR*G
	RBS positions (H3 numbering)										
	Q226L	226	L	L	L	I	L	L	Q	L	L
	G228S	228	G	G	G	G	G	G	G	G	G
	Glycosylation motifs		30NGTK, 46NATE, 249NDTV, 421NWTR, 493NNTY	30NGTK, 46NATE, 249NDTV, 421NWTR, 493NNTY	30NGTK, 46NATE, 249NDTV, 421NWTR, 493NNTY	30NGTK, 46NATE, 249NDTV, 421NWTR, 493NNTY	30NGTK, 46NATE, 249NDTV, 421NWTR, 493NNTY	30NGTK, 46NATE, 249NDTV, 421NWTR, 493NNTY	30NGTK, 46NATE, 249NDTV, 421NWTR, 493NNTY	30NGTK,46NATE, 249NDTV, 421NWTR, 493NNTY	30NGTK, 46NATE, 249NDTV, 421NWTR, 493NNTY
NA	Stalk		69-73 Del	69-73 Del	69-73 Del	69-73 Del	69-73 Del	69-73 Del	69-73 Del	69-73 Del	69-73 Del
	Antiviral resistance (Oseltamivir)										
	E120V	120	E	E	E	E	E	E	E	E	E
	H276Y	276	H	H	H	H	H	H	H	H	H
	R294K	294	R	R	R	R	R	R	K	R	R
PB2	Enhanced polymerase activity and increased virulence in mice										
	L89V	89	V	V	V	V	V	V	V	V	V
	Q591K	591	Q	Q	Q	Q	Q	Q	Q	Q	Q
	E627K	627	E	E	E	E	E	K	K	K	E
	D701N	701	D	D	D	N	D	D	D	D	D
PB1	H5 virus transmissible among ferrets										
	H99Y	99	H	H	H	H	H	H	H	H	H
	I368V	368	V	V	V	I	V	V	I	V	V
PB1-F2	Full length		90 aa	90 aa	90 aa	90 aa	90 aa	90 aa	90 aa	90 aa	99a
M1	Increased virulence in mice										
	N30D	30	D	D	D	D	D	D	D	D	D
	T215A	215	A	A	A	A	A	A	A	A	A
M2	Antiviral resistance (amantadine)										
	S31N	31	N	N	N	N	N	N	N	N	N
NS1	Increased virulence in mice										
	P42S	42	S	S	S	S	S	S	S	S	S
	V192I	192	V	I	I	V	V	V	V	V	V
	Decreased virulence in mice	218	Del	Del	Del	Del	Del	Del	Del	Del	Del
NP	A22T	22	T	T	A	T	A	A	A	A	A
	R246 K	246	K	K	K	R	R	R	R	R	R
	V280A	280	V	A	A	V	V	V	V	V	V
	N321S	321	N	N	N	S	N	N	N	N	N
	D375E	375	D	D	D	D	D	D	E	D	D
PA	I308V	308	I	I	I	V	I	I	I	I	I
	T618K	618	T	T	T	T	T	T	T	T	T

Single letters refer to the amino acid (Aa) found in the noted protein at a specific site. *The numbering starts with the first condon of Methionine for these proteins.

**Table 3:** **Protein sequence identity of three confirmed cases infected with avian influenza A (H7N9) virus, Zhejiang Province, China**

Gene	Family cluster isolates			Human spondaic isolates				H7N9 vaccine candidate isolate	Animal isolates
	Index case	Case 2	Case 3	A/Zhejiang/1/2013	A/Zhejiang/22/2013	A/Zhejiang/01/2014	A/Shanghai/1/2013	A/Anhui/1/2013	A/chicken/Zhejiang/SD019/2013
HA (%)	-	100	100	99.2	99.3	99.6	98.9	99.6	99.6
NA (%)	-	100	100	99.1	99.4	99.8	99.2	99.7	99.5
PB2 (%)	-	100	100	96.3	96.5	98.5	96.2	96.6	96.4
PB1 (%)	-	99.9	99.9	98.9	99.3	99.6	98.9	99.3	99.3
M (%)	-	100	100	99.7	97.6	99.7	97.8	97.7	97.7
PA (%)	-	100	100	99.6	99.4	99.4	99.6	99.7	99.7
NS1 (%)	-	99.9	99.9	98.8	98.6	99.5	98.9	99.2	99.2
NP (%)	-	99.6	99.6	99.2	99.6	99.2	98.4	99.4	99.4

Notation: − No available. The homology of the other isolates was based on the comparisons of the isolate from index case.

### Epidemiology findings

The Index case, his daughter (Case 2), his wife (Case 3), and his son-in-law (the husband of Case 2) lived in separate rooms of one large house with three floors. There were no domestic animals and birds within the home or in the immediate vicinity of the home. However, two neighboring families located 100 meters and 500 meters respectively from the cases’ homebred ducks and chickens, and there were a several free-range domestic poultry in the village.

The Index case and Case 3 worked in the LBM (A1 market), selling vegetables and bird eggs between 4 am and 7 pm during the two weeks prior to the illness onset in the Index case. Furthermore, two weeks before the illness onset, the Index case had visited a wholesale LBM (D1 market) to buy vegetables twice per week (each time he stayed there for 3 hours). The last known exposure date of the Index case in A1 market was January 12 (15 hours), and the last exposure date of Case 2 to A1 market was on January 17 for around three hours. In total. Case 3 had been exposed to the live bird market on three occasions for a total of 22 hours from January 17–20 as follows: Case 3 visited D1 market for a total of three hours between January 16–18 and she worked in A1 market for 8, 10 and, 1 hours on January 17, 18, and 20, respectively.

The Index case became ill on January 13 and was admitted to hospital on January 17. Between January 13 and 17, Cases 2 and 3 lived together with the Index case in one house. Furthermore, Case 3 and Index case had very close contact between January 13 and 17, sleeping together in one room. Case 2 had three hours face-to-face contact with the Index case on January 14. On January 17, Case 2 provided bedside care in the hospital for the Index case for approximately 12 hours. Between January 17 and 19, Cases 2 and 3 provided bedside care to the Index case in hospital without any personal protective equipment for approximately 30 hours and 7 hours, respectively, including washing, cleaning his body, change his clothes and disposing urine and feces of the Index case. During this period, the Index case had high fevers (39.9°C), frequent coughing, and extensive sputum production. After the Index case had been confirmed H7N9 infection on January 19, he moved to ICU for treatment and isolation. Cases 2 and 3 visited the Index case for four hours on January 19, wearing facemasks. Case 3 had frequent close contact with Case 2 during the funeral ceremony of the Index case on January 21–22. Case 3 visited Case 2 on the January 23 when Case 2 was hospitalized with mild symptoms; Case 3 wore a facemask during this visit. A summary of the cases’ exposure to each other was shown in Table 4.

**Table 4:** **Detailed exposure of case 3, case 2 to the index case confirmed H7N9 before the onset of illness**

Date	Activities	Exposure details	Duration (h)	Index case		Case 2		Case 3	
				Days after onset of illness (days)	Signs and symptoms	Days before onset of illness (days)	Personal protective equipment used	Days before onset of illness(days)	Personal protective equipment used
Jan 14th	Index case were consulted with Case 2	Case 2 exposed to Index case <1 m	3	1	Fever with 39.6°C	6	No		
Jan 16th	1. Index case was consulted with Case 2	1. Case 2 exposed to Index case <1 m	12	3	Fever with 40°C, short of breath and developing pneumonia	4	No		
	2. Case 2 provided bedside care for Index case in hospital	2. Slept on Index case’s bed, hugged him, washed Index case’ body with bare hands and helped to change clothes							
Jan 17-18th	Case 2 and Case 3 provided bedside care for Index case in hospital	1. Case 2 washed Index case body and changed his cloth	Case 2 and 3 were exposure for 30 and 7 hrs respectively	4	Fever with 39.8°C; cough; nausea; short of breath	3	No	5	No
		2. Case 2 exposed to Index case < 1 m							
		3. Case 3 cleaned, washed the Index case’ body and secretion and changed his cloth							
Jan 19th	1.Case 2 visiting to Index case	1.Case 2 exposed to Index case < 1 m	Case 2 and 3 were exposure for 4 hrs respectively	6	Constant fever and be hospitalized in ICU	1	Yes	5	Yes
	2.Case 3 visiting to Index case	2.Case 3 exposed to Index case < 1 m							
Jan 22nd	Index case’s Funeral	Case 3 exposed to Case 2 < 1 m	4	9	Died	2	No	1	No
Jan 23rd	Case 3 visiting to Case 2	Case 3 exposed to Case 2 < 1 m	2	10	Died	3	No	1	Yes

Note: ICU = Intensive care unit.

The results of RT-PCR assay of environment samples were listed as follows: 4 swabs of chicken and duck eggs from the Index case working site (A1 market) were H7N9 negative; 3 of 5 environmental samples from secondary live wet market (B1 market, a wholesale for A1 market) were positive for H7N9; 1 of 2 sewage samples from C1 market (located nearby B1 market) were positive for H7N9; 10 of 20 environmental samples taken from D1 market through routine avian influenza surveillance were H7N9 positive; 12 environmental samples from the area where neighbors were breeding poultry were all H7N9 negative; 11 of 26 environmental samples from different live birds markets under routine surveillance in Xiaoshan district were H7N9 positive during January 2014 (source: unpublished data from the Zhejiang Avian Surveillance System, Additional file 1: Figure S3).

### Contacts investigation

None of the 25 close contacts developed acute respiratory symptoms during the seven days surveillance period. Throat swabs collected from all twenty-five close contacts on January 24 were negative for influenza A/H7N9 virus by rRT-PCR, and all serum samples tested negative for H7N9 antibodies (titer < 1:40) by microneutralization and horse red-blood-cell HI assays (see Table 5). No close contacts were reported taking Oseltamivir chemoprophylaxis.

**Table 5:** **Type of exposure and sera collection of 25 close contacts**

Detail	Only exposed to Index case (N = 0)	Only exposed to Case 2 (N = 0)	Only exposed to Case 3 (N = 1)	Exposed to both Index case and Case 2 (N = 10)	Exposed to both Index case and Case 3 (N = 0)	Exposed to both Case 2 and Case 3 (N = 13)	Exposed to three cases (N = 1)	Total (N = 25)
Age (years)	-	-	35	55	-	45	25	47.5
Sex (male)	-	-	1	6	-	9	1	16
Exposure birds								
Contact with well-appearing poultry*	-	-	0	0	-	6	0	6
Contact with sick or dead poultry	-	-	0	0	-	0	0	0
Visited wet poultry market	-	-	0	8	-	4	0	12
Types of contact with H7N9 cases								
Provided direct care	-	-	0	0	-	0	0	0
Close physical contact	-	-	0	0	-	0	1	1
Exposed to case <1 m	-	-	0	2	-	13	0	15
Recalled case coughing or sneezing	-	-	0	0	-	0	0	0
Contact with respiratory or fecal secretions	-	-	0	0	-	0	1	1
Mean (range) duration of exposure to cases (h)	-	-	0	1	-	3 h	8 h	11 h
Oseltamivir used for chemoprophylaxis	No	No	No	No	No	No	No	No
Personal protection equipment	No	No	No	No	No	No	No	No
N95 respirator	No	No	No	No	No	No	No	No
Glasses	No	No	No	No	No	No	No	No
Surgical mask	No	No	No	No	No	No	No	No
Face shield	No	No	No	No	No	No	No	No
Gloves	No	No	No	No	No	No	No	No
Gowns	No	No	No	No	No	No	No	No
Febrile respiratory symptoms	0	0	0	0	0	0	0	0
Time from last exposure to only convalescent serum collection (days)	-	-	1 day	2 days	-	2 days	1 day	1 ~ 2 days
Time from last exposure to respiratory specimens collection (days)	-	-	1 day	2 days	-	2 days	1 day	1 ~ 2 days

Note: Data are median (IQR) or n (%). *Including direct contact (touching), preparation, cooking, and consumption of well-appearing poultry.

## Discussion

Here we describe a family cluster of three confirmed cases of H7N9 virus infection, involving a fatal Index case, his wife and daughter (both survived). The Index case presented with severe pneumonia and died of ARDS and multi-organ failure. The presence of chronic diseases has been associated with an increased risk of hospitalization with H7N9 virus infection [22], and the Index case had pre-existing diabetes, which requires oral anti-diabetic medication. Another factor that may have played a role in the severity of disease was the late diagnosis of H7N9 virus infection and the late commencement of anti-viral therapy. The efficacy of neuraminidase inhibitors (NAIs) in reducing the risk of mild influenza infection progressed to severe illness has not been fully assessed in randomized controlled trials; however, observational data suggest that early treatment with NAIs of hospitalized patients with influenza infection is associated with better outcomes [23]. The other two cases were previously reported healthy, and presented with lower viral loads and mild symptoms that did not progress. Both patients received early antiviral treatment, but it is not possible to determine whether the lack of clinical progression was result from antiviral treatment or as a consequence of a naturally indolent course [24]-[26]. Since there were no functionally important differences in the genotype of the virus infecting the three cases, viral virulence is not likely to contribute the differential severity.

WHO evaluates all clusters of human cases of non-seasonal influenza virus to determine whether human-to-human transmission or common exposure to infected animals or contaminated environments may have occurred [19]. The homology of all eight gene segments was between 99.6 ~ 100%, suggesting it was either a common source exposure or a person-to-person transmission. Whilst all three individuals were exposed to potentially contaminated market environments within a putative maximum incubation period of 7 days, Case 2 and Case 3 had extensive unprotected exposure to the Index cases when he was ill. We believe that most likely explanation for this family cluster is that the Index case was infected from the live bird market, and the virus was transmitted directly from the Index case to his daughter and his wife. Several reasons could explain for this conclusion, as follows: (1) 7 days prior to illness onset in the Index case, he had not been in contact with any people with a febrile illness and other confirmed cases, but was frequently exposed to the A1 live bird market for 9 hours daily and to the D1 secondary live bird market. Although the A1 market was H7N9-negative based on the environments samples collected on January 24, 2014, the samples from wholesale market B1 that supplied A1 market were H7N9 positive. Furthermore, 42.30% (11/26) of environments samples from different live bird markets under routine surveillance in Xiaoshan district during the same period were H7N9 positive (Source: unpublished data from the Zhejiang Avian Surveillance System); (2) Case 2 stayed with the Index case and provided beside bed medical care frequently on the January 14, 16, and 17–19. She had close unprotected contact with the Index case for cleaning and washing his body on January 18 without any personal protection when the Index case had severe symptoms such as high fever and cough. Although Case 2 had visited the A1 live bird market for three hours in three days prior to her illness onset, she reported no direct contact with live birds or poultry products. (3) There were multiple potential sources of infection for Case 3, including the Index case, the live market A1, and Case 2. However, the Index case and Case 3 shared the same room every day and worked closely together after the illness onset in the Index case. Most importantly, Case 3 provided beside bed care to the Index case including washing his body, dealing with his secretions, and changing his clothes for him, without any personal protective equipment. The day numbers between the onset of illness in the Index case and the onset of illness in the secondary cases (the serial interval) was 7 and 10 days, respectively. The incubation period reported by Cowling et al. is 3 days and that reported by Huang et al. is 7.5 days [27],[28].

Furthermore, sequence analysis showed that four strains isolated from the three cases were genetically similar to each other. All four isolates possessing amino acids Q226L and G228S in the HA segment were associated with increased affinity for human receptors (α-2, 6-linked sialo-saccharides) [29]. virus from all three cases possessed P42S in NS and E627K and D701N in PB2 (which were associated with increased virulence in mice) and I368V and H99Y in PB1 (which was associated with aerosol transmission of avian virus between ferrets) [7],[8]. There were only two amino differences (V192I in NS and V280A in NP) between the virus infecting the Index case and the secondary cases. Those two mutations are not associated with any known functional change. Therefore, field investigation and H7N9 full genomics analyses supported the secondary cases acquired infection most likely from the Index case. Person-to-person transmission of H7N9 has been reported [30],[31]. Previous animal experiments (ferrets, mice, and pigs) also indicate that H7N9 virus possess the capability to bind to both avian and human receptors and it might be transmissible by respiratory droplets under certain conditions [12],[15].

Our findings indicate that the virus has not gained the ability for efficient sustained transmission from person to person [12]. In this study, four close contacts and 21 frequent contacts were negative for H7N9 infection by HI testing and RT-PCR. Although the husband of Case 2 had close contact with the Index case, Case 2, and Case 3 without any personal protective equipment, he showed no evidence of infection with the H7N9 virus.

There were several limitations in this paper. Firstly, H7N9 positive samples in environmental or bird samples were not found from A1 live bird market where the Index case and Case 3 were working. Secondly, the full genetic sequence of H7N9 virus detected in the environment and live birds could not obtained. Thus it is not able to compare human, avian, and environmental strains.

On the basis of experiences of controlling of H5N1 and H7N9 virus, continued risk assessment, surveillance, and vigilance are required. A high degree of clinical awareness is necessary for people with possibility of H7N9 infection, especially for health workers who are occupationally exposed to poultry and for people with respiratory illness following recent contact with live poultry or live bird markets [32],[33].

## Conclusions

Here we report a largest size of the family cluster with confirmed H7N9 in China, in which the Index case was fatal while the secondary cases were mild. In the term of an infectious source, The Index case was infected from a live bird market while the Index case infected the two secondary cases during unprotected frequent exposure. This family cluster supported that the transmission of avian influenza A/H7N9 was limited and not sustained.

### Availability of supporting data

All of H7N9 referred isolates in additional materinals were download from GenBank (http://www.ncbi.nlm.nih.gov/nuccore/?term=H7N9++AND+CHINA) and The Global Initiative on Sharing All Influenza Data (GISAID) (http://platform.gisaid.org/epi3/frontend#50dda5).

## Additional file

## Electronic supplementary material

Additional file 1: **Figure S1.** Family pedigree showing three H7N9 affected individuals and their close contacts. **Figure S2.** Phylogenetic analysis of six segments (MP, NP, NS, PA, PB1, and PB2) from the four H7N9 isolates in three confirmed cases of a family cluster in Hangzhou, Zhejiang Province, China, in January of 2014. **Figure S3.** The geographical distribution of three H7N9 confirmed cases and related with live bird market in Xiao Shan district, Hangzhou, Zehjiang Province in 2014. (DOCX 381 KB)

Below are the links to the authors’ original submitted files for images.

Authors’ original file for figure 1

Authors’ original file for figure 2

Authors’ original file for figure 3

Authors’ original file for figure 4

## Abbreviations

Aa: Acid amino
ARDS: Adult respiratory distress syndrome
BSL: Biosafety level
CDC: Centre for disease control and prevention
HA: Hemagglutinin
ICU: Intensive care unit
IQR: Interquartile range
LBM: Live bird market
M1: Matrix protein 1
M2: Matrix protein 1
NA: Neuraminidase
NP: Nucleoprotein
NS1: Nonstructural protein 1
NS2: Nonstructural protein 2
PA: Polymerase protein
PB1: Polymerase basic 1
PB2: Polymerase basic 2
RT-PCR: Real-time reverse-transcriptase-polymerase-chain-reaction assay
SARI: Severe acute respiratory illness

## References

[1] Human infections with avian influenza A(H7N9) virus [http://www.who.int/influenza/human_animal_interface/influenza_h7n9/riskassessment_h7n9_2Oct14.pdf?ua=1]

[2] Jernigan DB, Cox NJ: H7N9: preparing for the unexpected in influenza. *Annu Rev Med* 2015, 66:361–71. doi: 10.1146/annurev-med-010714-112311. Epub 2014 Oct 29., 10.1146/annurev-med-010714-112311 25386931

[3] Abdel-Ghafar AN, Chotpitayasunondh T, Gao Z, Hayden FG, Nguyen DH, de Jong MD, Naghdaliyev A, Peiris JS, Shindo N, Soeroso S, Uyeki TM, Writing Committee of the Second World Health Organization Consultation on Clinical Aspects of Human Infection with Avian Influenza AV (2008). Update on avian influenza A (H5N1) virus infection in humans. N Engl J Med.

[4] Liu SL, Zhang ZR, Wang C, Dong Y, Cui LB, Yang XH, Sun Z, Wang J, Chen J, Huang RJ, Miao F, Ruan B, Xie L, He HX, Deng J (2010). 2009 pandemic characteristics and controlling experiences of influenza H1N1 virus 1 year after the inception in Hangzhou, China. J Med Virol.

[5] Mok CK, Lee HH, Lestra M, Nicholls JM, Chan MC, Sia SF, Zhu H, Poon LL, Guan Y, Peiris JS (2014). Amino acid substitutions in polymerase basic protein 2 gene contribute to the pathogenicity of the novel A/H7N9 influenza virus in mammalian hosts. J Virol.

[6] Cui L, Liu D, Shi W, Pan J, Qi X, Li X, Guo X, Zhou M, Li W, Li J, Haywood J, Xiao H, Yu X, Pu X, Wu Y, Yu H, Zhao K, Zhu Y, Wu B, Jin T, Shi Z, Tang F, Zhu F, Sun Q, Wu L, Yang R, Yan J, Lei F, Zhu B, Liu W (2014). Dynamic reassortments and genetic heterogeneity of the human-infecting influenza A (H7N9) virus. Nat Commun.

[7] Chen E, Chen Y, Fu L, Chen Z, Gong Z, Mao H, Wang D, Ni MY, Wu P, Yu Z, He T, Li Z, Gao J, Liu S, Shu Y, Cowling BJ, Xia S, Yu H: Human infection with avian influenza A(H7N9) virus re-emerges in China in winter 2013. *Euro Surveill* 2013, 18(43). pii: 20616., 10.2807/1560-7917.es2013.18.43.20616 24176616

[8] Bao CJ, Cui LB, Zhou MH, Hong L, Gao GF, Wang H (2013). Live-animal markets and influenza A (H7N9) virus infection. N Engl J Med.

[9] Belser JA, Gustin KM, Pearce MB, Maines TR, Zeng H, Pappas C, Sun X, Carney PJ, Villanueva JM, Stevens J, Katz JM, Tumpey TM (2013). Pathogenesis and transmission of avian influenza A (H7N9) virus in ferrets and mice. Nature.

[10] Richard M, Schrauwen EJ, de Graaf M, Bestebroer TM, Spronken MI, van Boheemen S, de Meulder D, Lexmond P, Linster M, Herfst S, Smith DJ, van den Brand JM, Burke DF, Kuiken T, Rimmelzwaan GF, Osterhaus AD, Fouchier RA (2013). Limited airborne transmission of H7N9 influenza A virus between ferrets. Nature.

[11] Xu L, Bao L, Deng W, Dong L, Zhu H, Chen T, Lv Q, Li F, Yuan J, Xiang Z, Gao K, Xu Y, Huang L, Li Y, Liu J, Yao Y, Yu P, Li X, Huang W, Zhao X, Lan Y, Guo J, Yong W, Wei Q, Chen H, Zhang L, Qin C (2014). Novel avian-origin human influenza A(H7N9) can be transmitted between ferrets via respiratory droplets. J Infect Dis.

[12] Song W, Wang P, Mok BW, Lau SY, Huang X, Wu WL, Zheng M, Wen X, Yang S, Chen Y, Li L, Yuen KY, Chen H (2014). The K526R substitution in viral protein PB2 enhances the effects of E627K on influenza virus replication. Nat Commun.

[13] Li J, Yu X, Pu X, Xie L, Sun Y, Xiao H, Wang F, Din H, Wu Y, Liu D, Zhao G, Liu J, Pan J (2013). Environmental connections of novel avian-origin H7N9 influenza virus infection and virus adaptation to the human. Sci China Life Sci.

[14] Li Q, Zhou L, Zhou M, Chen Z, Li F, Wu H, Xiang N, Chen E, Tang F, Wang D, Meng L, Hong Z, Tu W, Cao Y, Li L, Ding F, Liu B, Wang M, Xie R, Gao R, Li X, Bai T, Zou S, He J, Hu J, Xu Y, Chai C, Wang S, Gao Y, Jin L (2014). Epidemiology of human infections with avian influenza A(H7N9) virus in China. N Engl J Med.

[15] Jie Z, Xie J, He Z, Song Y, Hu Y, Li F, Shen Y, Shi J, He Y, Huang Q, Gu Y, Bai C (2013). Family outbreak of severe pneumonia induced by H7N9 infection. Am J Respir Crit Care Med.

[16] Song R, Pang X, Yang P, Shu Y, Zhang Y, Wang Q, Chen Z, Liu J, Cheng J, Jiao Y, Jiang R, Lu L, Chen L, Ma J, Li C, Zeng H, Peng X, Huang L, Zheng Y, Deng Y, Li X (2014). Surveillance of the first case of human avian influenza A (H7N9) virus in Beijing, China. Infection.

[17] Qi X, Qian YH, Bao CJ, Guo XL, Cui LB, Tang FY, Ji H, Huang Y, Cai PQ, Lu B, Xu K, Shi C, Zhu FC, Zhou MH, Wang H (2013). Probable person to person transmission of novel avian influenza A (H7N9) virus in Eastern China, 2013: epidemiological investigation. BMJ.

[18] Liu T, Bi Z, Wang X, Li Z, Ding S, Bi Z, Wang L, Pei Y, Song S, Zhang S, Wang J, Sun D, Pang B, Sun L, Jiang X, Lei J, Yuan Q, Kou Z, Yang B, Shu Y, Yang L, Li X, Lu K, Liu J, Zhang T, Xu A (2014). One family cluster of avian influenza A(H7N9) virus infection in Shandong, China. BMC Infect Dis.

[19] Watanabe T, Kiso M, Fukuyama S, Nakajima N, Imai M, Yamada S, Murakami S, Yamayoshi S, Iwatsuki-Horimoto K, Sakoda Y, Takashita E, McBride R, Noda T, Hatta M, Imai H, Zhao D, Kishida N, Shirakura M, de Vries RP, Shichinohe S, Okamatsu M, Tamura T, Tomita Y, Fujimoto N, Goto K, Katsura H, Kawakami E, Ishikawa I, Watanabe S, Ito M (2013). Characterization of H7N9 influenza A viruses isolated from humans. Nature.

[20] Zhu H, Wang D, Kelvin DJ, Li L, Zheng Z, Yoon SW, Wong SS, Farooqui A, Wang J, Banner D, Chen R, Zheng R, Zhou J, Zhang Y, Hong W, Dong W, Cai Q, Roehrl MH, Huang SS, Kelvin AA, Yao T, Zhou B, Chen X, Leung GM, Poon LL, Webster RG, Webby RJ, Peiris JS, Guan Y, Shu Y (2013). Infectivity, transmission, and pathology of human-isolated H7N9 influenza virus in ferrets and pigs. Science.

[21] Gao R, Cao B, Hu Y, Feng Z, Wang D, Hu W, Chen J, Jie Z, Qiu H, Xu K, Xu X, Lu H, Zhu W, Gao Z, Xiang N, Shen Y, He Z, Gu Y, Zhang Z, Yang Y, Zhao X, Zhou L, Li X, Zou S, Zhang Y, Li X, Yang L, Guo J, Dong J, Li Q (2013). Human infection with a novel avian-origin influenza A (H7N9) virus. N Engl J Med.

[22] Wang C, Yu H, Horby PW, Cao B, Wu P, Yang S, Gao H, Li H, Tsang TK, Liao Q, Gao Z, Ip DK, Jia H, Jiang H, Liu B, Ni MY, Dai X, Liu F, Van Kinh N, Liem NT, Hien TT, Li Y, Yang J, Wu JT, Zheng Y, Leung GM, Farrar JJ, Cowling BJ, Uyeki TM, Li L (2014). Comparison of patients hospitalized with influenza A subtypes H7N9, H5N1, and 2009 pandemic H1N1. Clin Infect Dis.

[23] Chan PK, Lee N, Zaman M, Adisasmito W, Coker R, Hanshaoworakul W, Gasimov V, Oner AF, Dogan N, Tsang O, Phommasack B, Touch S, Bamgboye E, Swenson A, Toovey S, Dreyer NA (2012). Determinants of antiviral effectiveness in influenza virus A subtype H5N1. J Infect Dis.

[24] Liu W, Zhu Y, Qi X, Xu K, Ge A, Ji H, Ai J, Bao C, Tang F, Zhou M (2013). Risk assessment on the epidemics of human infection with a novel avian influenza A (H7N9) virus in Jiangsu Province, China. J Biomed Res.

[25] Uyeki TM, Cox NJ (2013). Global concerns regarding novel influenza A (H7N9) virus infections. N Engl J Med.

[26] Lu S, Xi X, Zheng Y, Cao Y, Liu X, Lu H (2013). Analysis of the clinical characteristics and treatment of two patients with avian influenza virus (H7N9). Biosci Trends.

[27] Cowling BJ, Jin L, Lau EH, Liao Q, Wu P, Jiang H, Tsang TK, Zheng J, Fang VJ, Chang Z, Ni MY, Zhang Q, Ip DK, Yu J, Li Y, Wang L, Tu W, Meng L, Wu JT, Luo H, Li Q, Shu Y, Li Z, Feng Z, Yang W, Wang Y, Leung GM, Yu H (2013). Comparative epidemiology of human infections with avian influenza A H7N9 and H5N1 viruses in China: a population-based study of laboratory-confirmed cases. Lancet.

[28] Huang Y, Xu K, Ren DF, Ai J, Ji H, Ge AH, Bao CJ, Shi GQ, Shen T, Tang FY, Zhu YF, Zhou MH, Wang H (2013). Probable longer incubation period for human infection with avian influenza A(H7N9) virus in Jiangsu Province, China. Epidemiol Infect.

[29] Xiong X, Martin SR, Haire LF, Wharton SA, Daniels RS, Bennett MS, McCauley JW, Collins PJ, Walker PA, Skehel JJ, Gamblin SJ (2013). Receptor binding by an H7N9 influenza virus from humans. Nature.

[30] Ungchusak K, Auewarakul P, Dowell SF, Kitphati R, Auwanit W, Puthavathana P, Uiprasertkul M, Boonnak K, Pittayawonganon C, Cox NJ, Zaki SR, Thawatsupha P, Chittaganpitch M, Khontong R, Simmerman JM, Chunsutthiwat S (2005). Probable person-to-person transmission of avian influenza A (H5N1). N Engl J Med.

[31] Kandun IN, Wibisono H, Sedyaningsih ER, Yusharmen, Hadisoedarsuno W, Purba W, Santoso H, Septiawati C, Tresnaningsih E, Heriyanto B, Yuwono D, Harun S, Soeroso S, Giriputra S, Blair PJ, Jeremijenko A, Kosasih H, Putnam SD, Samaan G, Silitonga M, Chan KH, Poon LL, Lim W, Klimov A, Lindstrom S, Guan Y, Donis R, Katz J, Cox N, Peiris M (2006). Three Indonesian clusters of H5N1 virus infection in 2005. N Engl J Med.

[32] Liu Q, Lu L, Sun Z, Chen GW, Wen Y, Jiang S (2013). Genomic signature and protein sequence analysis of a novel influenza A (H7N9) virus that causes an outbreak in humans in China. Microbes Infect.

[33] Yu H, Cowling BJ, Feng L, Lau EH, Liao Q, Tsang TK, Peng Z, Wu P, Liu F, Fang VJ, Zhang H, Li M, Zeng L, Xu Z, Li Z, Luo H, Li Q, Feng Z, Cao B, Yang W, Wu JT, Wang Y, Leung GM (2013). Human infection with avian influenza A H7N9 virus: an assessment of clinical severity. Lancet.

