# Supplementary material for: A family cluster of three confirmed cases infected with avian influenza A (H7N9) virus in Zhejiang Province of China
Source: BMC Infect Dis. 2014 Dec 31;14:698. doi: 10.1186/s12879-014-0698-6 (PMC4304124; doi:10.1186/s12879-014-0698-6)
Supplement: Supplementary file 1 — Additional file 1: Figure S1. Family pedigree showing three H7N9 affected individuals and their close contacts. Figure S2. Phylogenetic analysis of six segments (MP, NP, NS, PA, PB1, and PB2) from the four H7N9 isolates in three confirmed cases of a family cluster in Hangzhou, Zhejiang Province, China, in January of 2014. Figure S3. The geographical distribution of three H7N9 confirmed cases and related with live bird market in Xiao Shan district, Hangzhou, Zehjiang Province in 2014. (DOCX 381 KB) [file 12879_2014_698_MOESM1_ESM.docx]

**Supplementary Figure 1. Family pedigree showing three H7N9 affected individuals and their close contacts**

Red color noted the death of H7N9; Green color noted survivor with H7N9 infection; Blue color: noted with close contacts; circle shape noted female; square shape noted male.

MP

NP

NS

PA

PB1

PB2

**Supplementary Figure 2. Phylogenetic analysis of six segments (MP, NP, NS, PA, PB1, and PB2) from the four H7N9 isolates in three confirmed cases of a family cluster in Hangzhou, Zhejiang Province, China, in January of 2014.**

Red color strains noted the isolates from three family cluster cases in 2014, while blue color noted the isolates from the confirmed spondaic cases in Zhejiang Province in 2013.


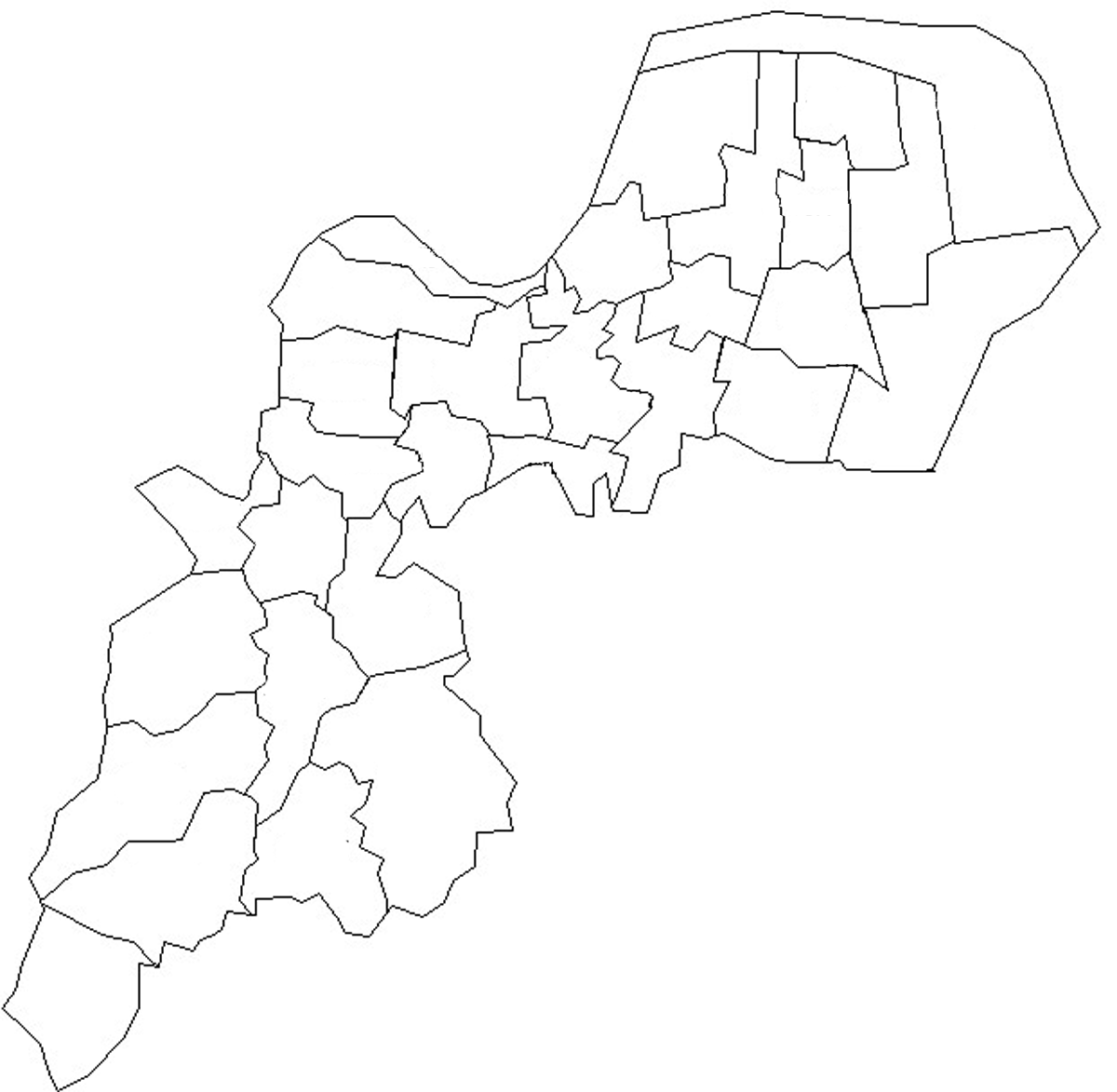


C1 market

**B1 market**

**A1 market**

**Supplementary Figure 3. The geographical distribution of three H7N9 confirmed cases and related with live bird market in Xiao Shan district, Hangzhou, Zehjiang Province in 2014**

H7N9 Positive live bird market; Three confirmed cases

H7N9 Positive countryside under H7N9 surveillance during January of 2014
