# Supplementary figures and images for: A family cluster of three confirmed cases infected with avian influenza A (H7N9) virus in Zhejiang Province of China
Source: BMC Infect Dis. 2014 Dec 31;14:698. doi: 10.1186/s12879-014-0698-6 (PMC4304124; doi:10.1186/s12879-014-0698-6)

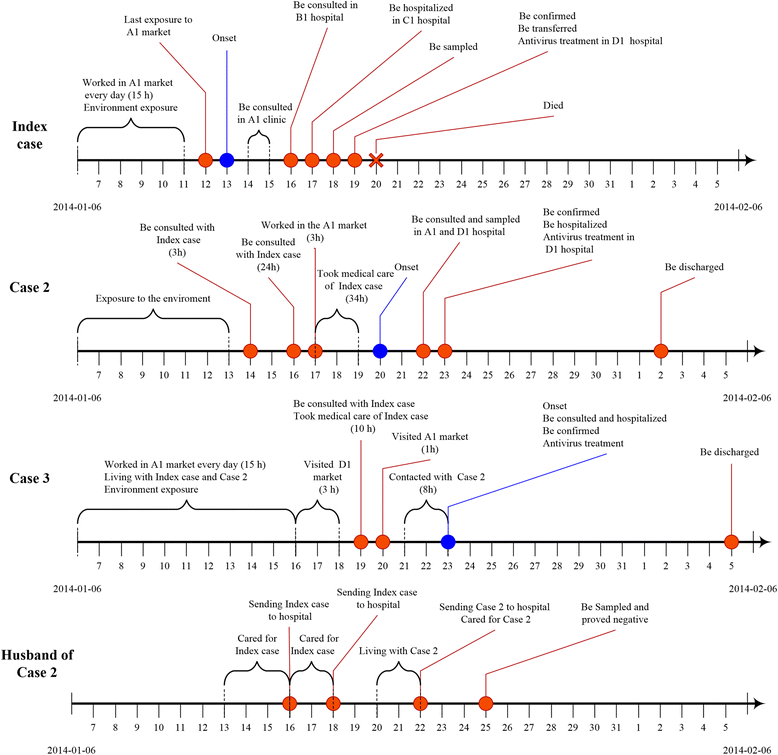

Supplement: Supplementary file 2 — Authors’ original file for figure 1 [file 12879_2014_698_MOESM2_ESM.gif]

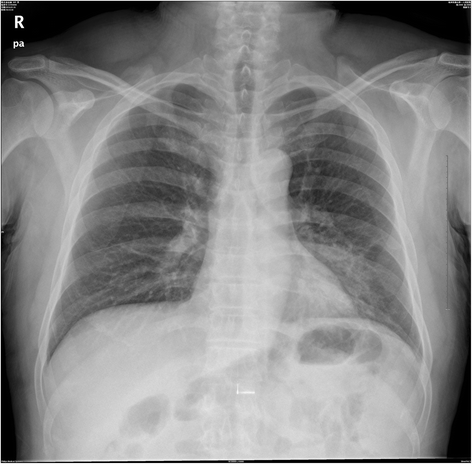

Supplement: Supplementary file 3 — Authors’ original file for figure 2 [file 12879_2014_698_MOESM3_ESM.gif]

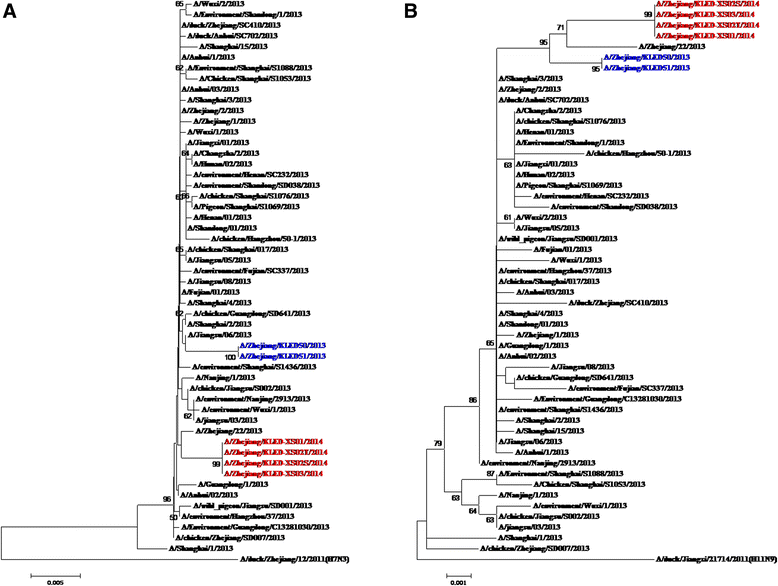

Supplement: Supplementary file 4 — Authors’ original file for figure 3 [file 12879_2014_698_MOESM4_ESM.gif]

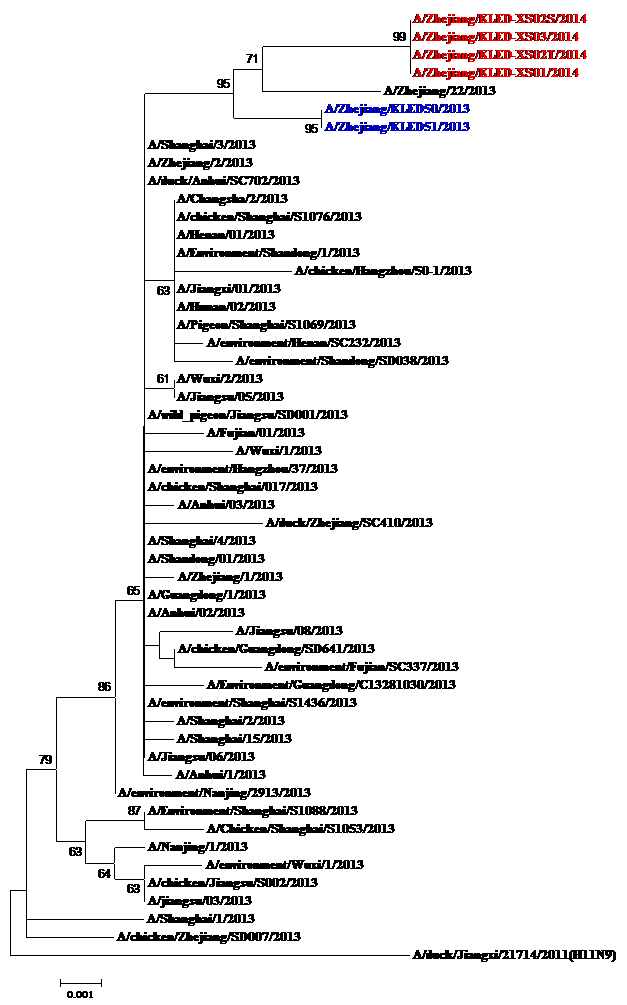

Supplement: Supplementary file 5 — Authors’ original file for figure 4 [file 12879_2014_698_MOESM5_ESM.tiff]
